# Anxiety, depression, and their associations with COVID-19-related knowledge, attitudes, and practices among healthcare professionals: a multinational cross-sectional survey

**DOI:** 10.1186/s40359-025-02783-0

**Published:** 2025-05-02

**Authors:** Wei-Cheng Lo, Yi-Chin Huang, Yi-Hao Weng, Mohammad Ainul Maruf, Chung Bui, Mei-Hui Lee, Kang-Yun Lee, Mai-Szu Wu, Ya-Wen Chiu, Hung-Yi Chiou

**Affiliations:** 1 Master Program in Applied Epidemiology, College of Public Health, Taipei Medical University, Taipei, Taiwan; 2 School of Public Health, College of Public Health, Taipei Medical University, Taipei, Taiwan; 3 Institute of Population Health Sciences, National Health Research Institutes, Miaoli, Taiwan; 4 Department of Pediatrics, Chang Gung Memorial Hospital, Chang Gung University College of Medicine, Taipei, Taiwan; 5 Faculty of Public Health, Universitas Muhammadiyah Jakarta, Jakarta, Indonesia; 6 Department of Health Communication and Education, Quang Ninh Provincial Center for Disease Control, Ha Long, Quang Ninh Vietnam; 7 Division of Medical Affairs, Department of Health, Ha Long, Quang Ninh Vietnam; 8 Division of Infectious Diseases, Department of Internal Medicine, Shuang Ho Hospital, Taipei Medical University, New Taipei, Taiwan; 9 Division of Pulmonary Medicine, Department of Internal Medicine, Shuang Ho Hospital, Taipei Medical University, New Taipei, Taiwan; 10 Division of Nephrology, Department of Internal Medicine, Shuang Ho Hospital, Taipei Medical University, New Taipei, Taiwan

**Keywords:** COVID-19, Health-care professionals, Anxiety, Depression, Multinational cross-sectional survey

## Abstract

**Objective:**

To investigate the levels of anxiety and depression (A&D) and the association with knowledge, attitudes, and practices among healthcare professionals (HCPs) in Taiwan, Indonesia, and Vietnam.

**Methods:**

A multinational cross-sectional survey was performed to collect data from 3,556 HCPs through online platforms. The Likert scale questionnaire covered sociodemographic factors, work-related information, and COVID-19-related domains, including knowledge, attitudes, practices, working conditions and availability of personal protective equipment, changes in work and life routines due to the COVID-19 pandemic, and experiences of A&D. Multiple logistic regression models were used to evaluate the potential impact of the aforementioned domains on the levels of A&D across and between countries.

**Results:**

Distinct A&D profiles emerged among the three countries. Taiwan exhibited higher A&D scores (average 2.31) than Vietnam (1.61) and Indonesia (1.93) (*p* < 0.001). Taiwan also showed elevated knowledge and attitudes scores. Consistent patterns were observed in responses on practices, working conditions, and pandemic impact on daily routines. Multivariate analysis showed that higher knowledge and attitudes scores were significantly associated with reduced A&D risk in Taiwan. Experiencing the greatest changes in work and daily routines was strongly linked to higher A&D risk, with adjusted odds ratios of 3.64 (95% CI: 1.41–9.45) in Indonesia, 4.13 (95% CI: 2.96–5.75) in Taiwan, and 5.14 (95% CI: 3.18–8.31) in Vietnam. Further analysis revealed that factors such as transportation, work dynamics, family time, dietary habits, and income level, but not leisure time, had varying impacts on A&D across the three countries.

**Conclusion:**

A&D and COVID-19-related knowledge, attitudes, and practices vary across countries. Thus, personalized support mechanisms and interventions are needed to address the diverse needs of HCPs within specific policy and country contexts.

**Supplementary Information:**

The online version contains supplementary material available at 10.1186/s40359-025-02783-0.

## Introduction

Coronavirus disease 2019 (COVID-19), emerging in Wuhan, China, in December 2019, quickly became a global crisis [1]. The World Health Organization (WHO) declared it a pandemic on March 11, 2020, following the announcement of a cluster of coronavirus-related pneumonia cases in Wuhan on January 9, 2020 [2]. The pandemic led to a rapid, uncontrollable escalation in global infections and deaths, posing significant challenges to public health and healthcare systems worldwide [3].

To control the spread of COVID-19, governments worldwide implemented various measures, such as imposing lockdowns and establishing isolation for individuals with suspected or confirmed infection. These measures profoundly affected the lives of individuals across the globe. Taiwan, with its comprehensive universal healthcare system (National Health Insurance), drew upon its experience in handling the 2003 SARS outbreak and responded swiftly to COVID-19 by enforcing strict border control measures, quarantine regulations, and widespread testing. Its sophisticated healthcare infrastructure and digital health management technology facilitated effective contact tracing and monitoring, leading to successful containment [4–6]. Similarly, Vietnam utilized its community-based primary healthcare model, with commune health stations playing a crucial role in proactive measures, community participation, and effective contact tracing to control the pandemic [7–9]. Public communication and awareness campaigns were pivotal in promoting adherence to COVID-19-related guidelines in Vietnam [10–12]. By contrast, Indonesia, implementing its Jaminan Kesehatan Nasional (JKN) program since 2014, faced significant challenges due to its decentralized healthcare system and diverse geographical landscape. Limited healthcare resources in remote regions posed challenges, and the decentralized governance system required coordination efforts [13, 14]. Each country’s response reflected its unique healthcare infrastructure, preventive medicine capabilities, and the variations in the development of the pandemic. The psychosocial effects of the pandemic on individuals, particularly healthcare professionals (HCPs), may vary across countries because of cultural factors and intervention measures. The implementation of strategies such as isolation, contact restrictions, and economic shutdowns markedly altered the psychosocial environment in COVID-19-affected countries, leading to psychological distress, poor coping behaviors, and noncompliance with public health measures [15]. The challenges arising from uncertainty about safety, unpredictable consequences, and misinformation [16] further increased the complexity of this situation.

Individuals’ understanding of and attitudes and behaviors toward infectious diseases—collectively known as knowledge, attitudes, and practices (KAP)—play pivotal roles in determining their adherence to disease control measures [17, 18]. Reducing the public’s fear of infectious diseases is crucial for effective transmission control [19]. Previous experiences with SARS suggest that individuals’ knowledge and attitudes toward a disease can influence panic and emotional responses in the general population [20, 21]. In the context of the COVID-19 pandemic, KAP considerably influenced individuals’ willingness to adopt different behaviors [22, 23]. Therefore, empirical studies on KAP are invaluable for designing effective interventions. Enhancing KAP can help address misconceptions and foster optimistic attitudes and appropriate practices [22–26]. Insights from the 2003 SARS outbreak indicate that infectious disease–related knowledge and practices are associated with a reduction in anxiety levels [27]. Thus, understanding these associations may help prevent complications due to disease spread. HCPs typically exhibited good knowledge, optimistic attitudes, and appropriate practices related to COVID-19 [28]. However, they were at elevated risks of anxiety and depression (A&D) because of the sudden and life-threatening nature of the disease [29–31]. Studies on SARS and Ebola epidemics have revealed that HCPs experienced extraordinary pressure and that SARS survivors, including HCPs, exhibited long-term psychiatric symptoms [31, 32]. Therefore, understanding the intricate associations between KAP and psychological responses in HCPs is crucial for devising effective frontline measures to overcome hurdles such as COVID-19.

To date, few studies have examined how COVID-19-related knowledge, attitudes, and practices are associated with A&D among HCPs across different countries. Thus, we conducted an online survey to identify these associations in HCPs in Taiwan, Vietnam, and Indonesia. Additionally, we further investigated whether these associations vary across the countries.

## Methods

### Study design, setting, and population

This cross-sectional survey included HCPs (age > 20 years) from Taiwan, Indonesia, and Vietnam. Eligible participants were HCPs engaged in clinical, allied health, public health, or community health services. Administrative personnel and non-hospital staff were excluded. Through cluster sampling, six teaching hospitals were purposively selected as the study sites, covering multiple clinical departments. These included Shuang Ho Hospital in northern Taiwan, three in Indonesia’s Jakarta (Cempaka Putih Jakarta Islamic Hospital, Syarif Hidayatullah Hospital, and Malahayati Islamic Hospital), and two in northern Vietnam (Quang Ninh Pediatric Hospital and Hanoi Lung Hospital). These hospitals were chosen for their comprehensive healthcare services, multidisciplinary workforce, and role in medical education, ensuring a diverse representation of healthcare professionals across different clinical settings. The total number of eligible participants from each hospital was verified through human resources departments for quality control. The response rates were 95.0% in Taiwan, 82.1% in Vietnam, and 60.1% in Indonesia.

Data were collected through an online survey, which was conducted from December 2021 to February 2022 in Taiwan (SurveyCake), from February 2021 to June 2021 in Indonesia (Google Forms), and from July 2021 to February 2022 in Vietnam (Google Forms). To ensure consistency, the questionnaire content, structure, and response options were identical across platforms. Assurances of data confidentiality and anonymity were provided to all participants. The participants were also offered paper-based questionnaires as an alternative means to accommodate their preferences. To ensure adequate sample representation and participation, a proactive approach was adopted for engaging potential participants. This involved liaising with the human resources department of each hospital and making direct contact with the heads or managers of each medical unit.

### Survey questionnaire

The questionnaire used in this study was adapted from previously validated surveys [22–26, 33–36], with modifications made to align with the study objectives and regional contexts. It collected data on diverse domains, such as sociodemographic factors (age, sex, and education level), work positions (managerial and teaching positions), and six COVID-19-related domains. These domains covered COVID-19-related knowledge and perception (9 items) [22–26, 33–36], attitudes (6 items) that reflect respondents’ interpretations and professional judgments regarding COVID-19 risks [22–24, 26, 34–36], and practices (10 items) with a focus on preventive behaviors [22–26, 34, 35]. Additional domains included experiences related to working conditions and personal protective equipment (PPE) availability during the pandemic (10 items) [33], COVID-19-related A&D (10 items) which are adapted from the items in the Kessler Psychological Distress Scale (K10) [37], and COVID-19-related changes in work and daily routines (6 items) [34, 35]. In addition, to reduce response bias, three reverse-scored items were included to encourage careful evaluation of the questions. For example, a reverse-scored item stated, ‘Children and youth do not need to take precautions against COVID-19.’ This approach improved the reliability and accuracy of the responses [38]. Also, all survey questions were initially developed in English. To ensure linguistic accuracy and cultural relevance, the questionnaire was then translated into Mandarin, Vietnamese, and Bahasa Indonesia by native speakers with expertise in medical terminology. Subsequently, a back-translation process was conducted by independent translators, who translated the items back into English to verify the accuracy and consistency of the translation.

To ensure the robustness of the questionnaire, a comprehensive pilot study and expert review were conducted before distribution. A panel of specialists in public health, epidemiology, infectious diseases, and clinical medicine evaluated the questionnaire’s content relevance and clarity, resulting in a content validity index of 0.95, well above the acceptable threshold of 0.75 [39]. Based on expert feedback, refinements were made to improve clarity and applicability. The questionnaire was then pilot-tested with 20 healthcare professionals to evaluate clarity and ease of completion. A test–retest reliability assessment at a one-week interval demonstrated a highly satisfactory reliability coefficient of 0.90, surpassing the acceptable threshold of 0.60 [40]. Also, the internal consistency of this questionnaire was assessed using Cronbach's Alpha, which yielded a value of 0.875. This indicates a high level of reliability, suggesting that the items on the questionnaire are consistently measuring the same underlying construct [41, 42]. A scoring framework was developed using a 5-point Likert scale. It was characterized by distinct gradations, with 1 denoting “strongly disagree” or “never/none of the time” and 5 denoting “strongly agree” or “always/all of the time.” Considering the presence of three diametrically opposite questions, responses were reverse-scored before statistical analyses. Elevated scores within this framework indicated improvements in COVID-19-related knowledge, attitudes, and practices. When assessing working conditions and PPE availability, elevated scores corresponded to enhanced workplace refinement and ample PPE supply. In the context of mental health assessment, increased scores indicated an increased incidence of A&D over the preceding 4-week period. Similarly, regarding the effects of COVID-19 on work and daily routines, higher scores indicated greater changes. The scores within each domain were summed to derive a total score, which was subsequently used to compute the mean score within each COVID-19-related domain.

### Statistical analyses

Descriptive statistics were used to summarize the participants’ demographic characteristics. Categorical data are presented in terms of absolute numerical counts and corresponding proportions. Between-country comparisons were performed using the chi-square test. When the A&D domain score exceeds (or includes) 20, it is categorized as having mild to severe A&D symptoms, while scores below 20 are classified as having none [43]. COVID-19-related factors were divided into four groups based on quartiles. Country-specific logistic regression analyses were performed to examine the associations between A&D and variables such as COVID-19-related knowledge, attitudes, practices, experiences with working conditions and PPE availability, and changes in work and daily routines. Multivariate logistic regression models were adjusted for covariates, including sociodemographic characteristics and work positions. In addition, a mixed-effects logistic regression model (fixed and random effects) was fitted using a cluster variable (country) to control for correlation within the country. All analyses were performed using SAS for Windows (version 9.4; SAS Institute Inc., Cary, NC, USA). Adjusted OR values and 95% confidence intervals were calculated. A two-tailed p-value of < 0.05 indicated statistical significance.

### Ethical considerations

This study was approved by the Joint Institutional Review Board of Taipei Medical University, Taiwan (N202008053); Health Research Ethics Commission of the Faculty of Public Health, University of Muhammadiyah Jakarta, Indonesia (10.340.B/KEPK-FKMUMJ/IX/2021); and Ethical Review Board for Biomedical Research, Hanoi University of Public Health, Vietnam (021–365/DD-YTCC). With the questionnaire, we provided an informed consent form outlining study objectives and clarifying data confidentiality. Anonymous participation was possible. This approach upheld ethical standards while maintaining research integrity.

## Results

A total of 3,556 participants from three countries were surveyed (1,673 from Taiwan, 402 from Indonesia, and 1,481 from Vietnam) (Table 1). Most respondents were women and aged < 40 years. In Taiwan, 58.6% of the respondents were educated till university or above. In Indonesia, 65.7% of the respondents had an education level below a bachelor’s degree. Most respondents in the three countries were nurses. In Vietnam, 26.3% of the respondents were physicians. Managerial roles were not common among the respondents in the three countries. In Taiwan, 27.1% of the respondents held teaching positions. In general, the respondents’ sociodemographic characteristics and work positions varied across the countries.

**Table 1:** Sociodemographic characteristics and work positions of healthcare professionals stratified by country

	**Indonesia** (*n* = 402)		**Taiwan** (*n* = 1,673)		**Vietnam** (*n* = 1,481)		
Variables	N	%	N	%	N	%	*p*-value
Sex							< 0.001
Male	83	20.6%	331	20.0%	414	27.9%	
Female	319	79.4%	1,322	80.0%	1,067	72.1%	
Age group (years)							< 0.001
20–29	131	32.6%	758	45.7%	408	27.5%	
30–39	160	39.8%	541	32.6%	799	53.8%	
40–49	90	22.4%	283	17.1%	217	14.6%	
> 50	21	5.2%	76	4.6%	60	4.1%	
Education							< 0.001
Below bachelor’s degree	264	65.7%	693	41.4%	651	44.0%	
Bachelor’s degree	60	14.9%	775	46.3%	608	41.1%	
Postgraduate degree	78	19.4%	205	12.3%	220	14.9%	
Clinical position							< 0.001
Physician	25	6.2%	255	15.3%	390	26.3%	
Nurse	262	65.2%	1,063	63.5%	658	44.3%	
Paramedic	115	28.6%	355	21.2%	436	29.4%	
Managerial position							0.0004
Yes	33	8.2%	133	8.0%	178	12.0%	
No	369	91.8%	1,540	92.0%	1,306	88.0%	
Teaching position							< 0.001
Yes	15	3.7%	453	27.1%	162	10.9%	
No	387	96.3%	1,220	72.9%	1,322	89.1%	

The sum of sample sizes for each demographic variable does not equal the total sample size is due to the missing values

**Fig. 1:**
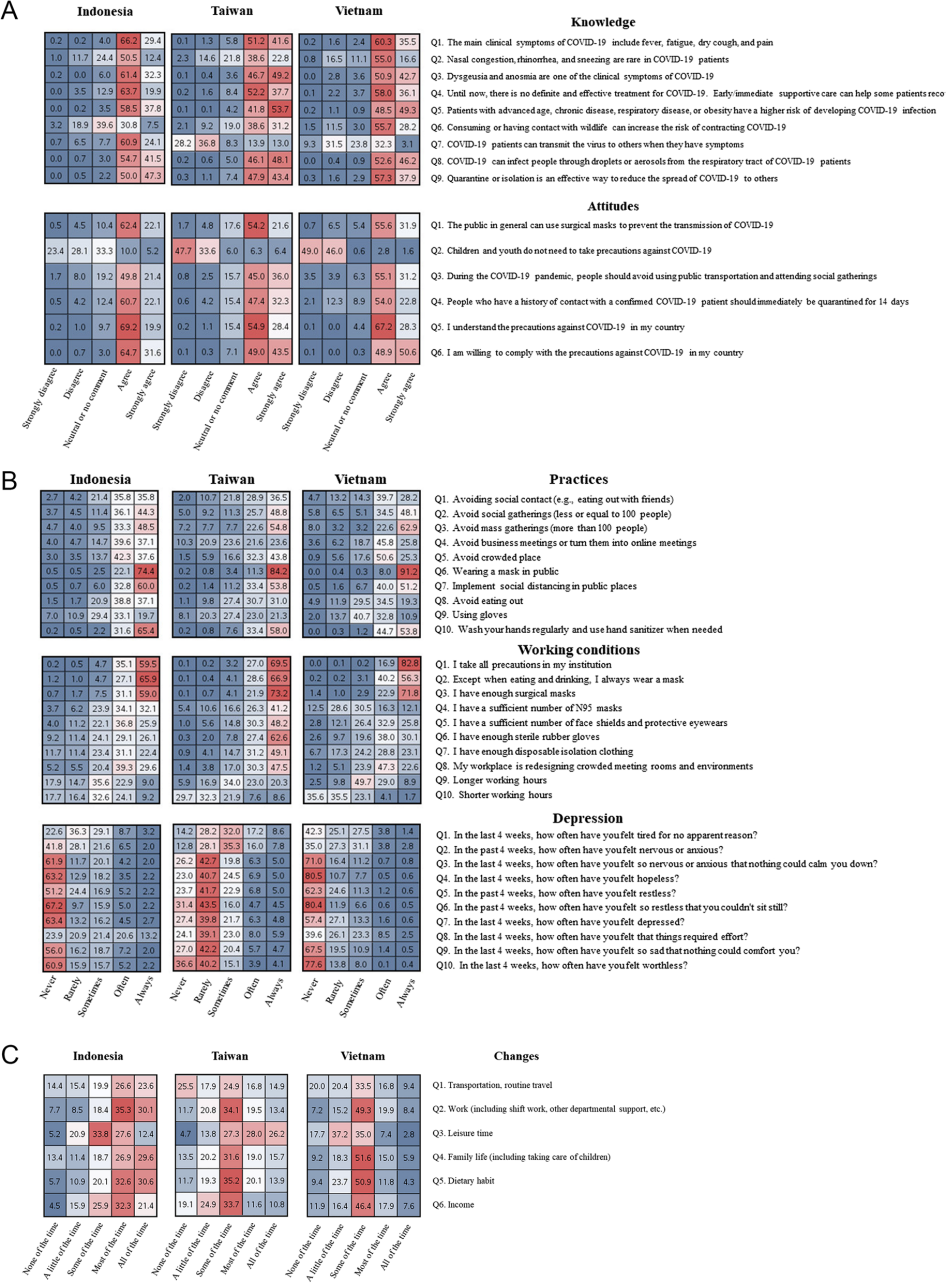
Heatmap of the survey responses of healthcare professionals stratified by country. **A** COVID-19-related knowledge and attitudes. **B** COVID-19-related practices, experiences related to working conditions and personal protective equipment availability during the pandemic, and COVID-19-related anxiety and depression. **C** COVID-19-related changes in work and daily routines. Cell values indicate response percentages

Figure 1 depicts the survey responses of HCPs stratified by country. COVID-19-related knowledge and attitudes exhibited similar trends across the three countries. However, in the knowledge domain, a notable between-country difference was observed in responses to Q7, with HCPs in Taiwan exhibiting relatively high levels of disagreement with the statement that COVID-19 patients can transmit the virus to others when they have symptoms. By contrast, HCPs in Vietnam maintained a neutral stance on this statement, whereas most HCPs in Indonesia were likely to agree with it. In the attitudes domain, contrasting responses were obtained for Q2 (children and youths do not need to take precautions against COVID-19). Most HCPs in Taiwan and Vietnam did not concur with this statement, whereas approximately one-third of all HCPs in Indonesia had a neutral stance (Fig. 1A). The respondents’ mean scores for the knowledge and attitudes domains varied significantly across the three countries. HCPs in Taiwan had the highest scores in both domains (*p* < 0.001 for the knowledge and attitudes domains).

The respondents’ responses to questions regarding COVID-19-related practices and experiences related to working conditions and PPE availability during the pandemic exhibited similar trends across the countries (*p* < 0.001 for the practices domain and the working condition and PPE availability domain). Furthermore, HCPs in Taiwan more frequently experienced A&D over the 4-week period before the survey than did HCPs in Vietnam or Indonesia. The mean A&D score was 2.31 for HCPs in Taiwan, 1.61 for HCPs in Vietnam, and 1.93 for HCPs in Indonesia (*p* < 0.001; Fig. 1B). Figure 1C depicts the COVID-19-related changes in the work and daily routines of HCPs stratified by country. HCPs in Indonesia typically perceived significant changes across various aspects of life, whereas those in Taiwan reported that their leisure time was most impacted. By contrast, most HCPs in Vietnam felt that the overall effect was not substantial (changes occurred in some aspects).

Table 2 presents the relationship between the other five COVID-19-related domains and the A&D level. Before controlling for other variables, the level of knowledge, attitudes, and practices did not significantly associate with the degree of A&D in Indonesia and Vietnam; however, participants with higher level (Q4) of knowledge (OR = 0.21; 95%CI 0.15–0.31), attitudes (OR = 0.25; 95%CI 0.18–0.35) and practices (OR = 0.76; 95% CI: 0.58–1.01, borderline significance) were less likely to have A&D in Taiwan. Regarding working conditions, A&D levels were negatively associated with working conditions in Taiwan, positively associated in Indonesia, and showed no significant association in Vietnam. A significant positive relationship was also noted between A&D and COVID-19-related changes in work and daily routines among HCPs across the three countries prior to adjusting for covariates.

**Table 2:** Univariate analysis for the associations of anxiety and depression with COVID-19-related domains across countries

**Domains**	**Indonesia** (*n* = 402)		**Taiwan** (*n* = 1,645)		**Vietnam** (*n* = 1,476)	
	OR	95% CI	OR	95% CI	OR	95% CI
Knowledge (K)						
Q1 (K < 3.66)	ref		ref		ref	
Q2 (3.66 ≦ K < 3.88)	0.90	[0.51, 1.57]	0.49	[0.33, 0.75]†	1.31	[0.89, 1.92]
Q3 (3.88 ≦ K < 4.22)	0.96	[0.59, 1.55]	0.36	[0.25, 0.52]†	1.36	[0.93, 1.98]
Q4 (K ≧ 4.22)	0.48	[0.13, 1.77]	0.21	[0.15, 0.31]†	1.44	[0.99, 2.09]
Attitudes (A)						
Q1 (A < 3.83)	ref		ref		ref	
Q2 (3.83 ≦ A < 4.16)	0.97	[0.59, 1.60]	0.58	[0.41, 0.82]†	0.71	[0.50, 1.02]
Q3 (4.16 ≦ A < 4.5)	0.70	[0.38, 1.29]	0.34	[0.25, 0.48]†	0.74	[0.51, 1.07]
Q4 (A ≧ 4.5)	0.89	[0.48, 1.65]	0.25	[0.18, 0.35]†	0.77	[0.54, 1.11]
Practices (P)						
Q1 (B < 3.7)	ref		ref		ref	
Q2 (3.7 ≦ P < 4.1)	1.23	[0.65, 2.31]	0.92	[0.68, 1.25]	0.86	[0.59, 1.24]
Q3 (4.1 ≦ P < 4.5)	0.82	[0.44, 1.54]	0.65	[0.48, 0.88]†	0.97	[0.69, 1.36]
Q4 (P ≧ 4.5)	0.57	[0.32, 1.03]	0.76	[0.58, 1.01]	1.03	[0.72, 1.49]
Working conditions (W)						
Q1 (W < 3.5)	ref		ref		ref	
Q2 (3.5 ≦ W < 3.9)	2.16	[1.20, 3.87]†	0.98	[0.66, 1.46]	1.14	[0.84, 1.55]
Q3 (3.9 ≦ W < 4.3)	1.81	[1.01, 3.24]*	0.66	[0.45, 0.95]*	1.10	[0.79, 1.52]
Q4 (W ≧ 4.3)	2.10	[1.17, 3.78]*	0.63	[0.45, 0.90]*	1.19	[0.81, 1.74]
Changes in work and daily routines (C)						
Q1 (C < 2.3)	ref		ref		ref	
Q2 (2.3 ≦ C < 3.0)	2.44	[0.86, 6.92]	1.50	[1.14, 2.02]†	2.79	[1.81, 4.31]†
Q3 (3.0 ≦ C < 3.5)	3.60	[1.34, 9.62]*	2.98	[2.19, 4.07]†	3.39	[2.19, 5.24]†
Q4 (C ≧ 3.5)	4.53	[1.84, 11.12]†	3.05	[2.27, 4.09]†	5.00	[3.14, 7.97]†

*OR* crude odds ratio, *95% CI *95% confidence intervals

*p < 0.05; †p < 0.01

Table 3 presents the results of a multivariate analysis examining the effects of sociodemographic factors, work positions, and five COVID-19-related domains on A&D across three countries. No significant collinearity was detected between the variables. In Indonesia, participants experiencing the greatest changes in work and daily routines, as well as those who have higher-score of working conditions (showing an increasing trend across quartiles), were more likely to report higher levels of A&D. In contrast, the results from Taiwan revealed that participants with higher level of knowledge, attitudes of COVID-19 and those holding postgraduate degree were significantly associated with a lower likelihood of having A&D disorders, with a clear decreasing trend across quartiles. However, no significant association between educational level and A&D was observed in Indonesia and Vietnam. Similar results from Indonesia, Taiwan, and Vietnam showed that greater changes in work and daily routines were consistently associated with elevated A&D levels, demonstrating an increasing trend across quartiles.

**Table 3:** Multivariate analysis for the effects of sociodemographic factors, work positions, countries, and five COVID-19-related domains on anxiety and depression

**Domains**	**Indonesia** (*n* = 402)		**Taiwan** (*n* = 1,645)		**Vietnam** (*n* = 1,476)	
	AOR	95% CI	AOR	95% CI	AOR	95% CI
Knowledge (K)						
Q1 (K < 3.66)	ref		ref		ref	
Q2 (3.66 ≦ K < 3.88)	0.93	[0.48, 1.81]	0.61	[0.39, 0.96]*	1.27	[0.85, 1.89]
Q3 (3.88 ≦ K < 4.22)	0.99	[0.53, 1.84]	0.56	[0.36, 0.85]†	1.40	[0.93, 2.11]
Q4 (K ≧ 4.22)	0.81	[0.19, 3.47]	0.43	[0.28, 0.67]†	1.38	[0.91, 2.11]
Attitudes (A)						
Q1 (A < 3.83)	ref		ref		ref	
Q2 (3.83 ≦ A < 4.16)	1.34	[0.75, 2.37]	0.77	[0.52, 1.13]	0.70	[0.48, 1.02]
Q3 (4.16 ≦ A < 4.5)	1.07	[0.50, 2.30]	0.42	[0.28, 0.62]†	0.68	[0.45, 1.01]
Q4 (A ≧ 4.5)	1.27	[0.55, 2.95]	0.39	[0.25, 0.59]†	0.65	[0.43, 0.98]*
Practices (P)						
Q1 (P < 3.7)	ref		ref		ref	
Q2 (3.7 ≦ P < 4.1)	1.14	[0.55, 2.37]	0.98	[0.70, 1.37]	0.82	[0.56, 1.22]
Q3 (4.1 ≦ P < 4.5)	0.70	[0.33, 1.50]	0.83	[0.58, 1.17]	0.91	[0.63, 1.30]
Q4 (P ≧ 4.5)	0.50	[0.23, 1.11]	1.16	[0.81, 1.66]	0.98	[0.66, 1.46]
Working conditions (W)						
Q1 (W < 3.5)	ref		ref		ref	
Q2 (3.5 ≦ W < 3.9)	2.96	[1.52, 5.78]†	1.06	[0.69, 1.64]	1.14	[0.83, 1.57]
Q3 (3.9 ≦ W < 4.3)	2.42	[1.21, 4.87]*	0.74	[0.49, 1.12]	1.02	[0.72, 1.45]
Q4 (W ≧ 4.3)	2.94	[1.44, 5.99]†	0.72	[0.47, 1.20]	1.12	[0.73, 1.70]
Changes in work and daily routines (C)						
Q1 (C < 2.3)	ref		ref		ref	
Q2 (2.3 ≦ C < 3.0)	1.72	[0.57, 5.23]	1.81	[1.31, 2.49]†	2.91	[1.87, 4.53]†
Q3 (3.0 ≦ C < 3.5)	2.74	[0.97, 7.75]	3.31	[2.36, 4.65]†	3.50	[2.24, 5.47]†
Q4 (C ≧ 3.5)	3.64	[1.41, 9.45]†	4.13	[2.96, 5.75]†	5.14	[3.18, 8.31]†
Sex						
Male	ref		ref		ref	
Female	0.83	[0.48, 1.45]	0.98	[0.70, 1.37]	1.06	[0.80, 1.42]
Age group (years)						
20–29	ref		ref		ref	
30–39	0.49	[0.29, 0.84]†	0.98	[0.75, 1.29]	0.80	[0.59, 1.08]
40–49	0.28	[0.14, 0.55]†	0.92	[0.65, 1.30]	1.35	[0.89, 2.05]
> 50	0.35	[0.11, 1.12]	0.67	[0.37, 1.22]	1.26	[0.64, 2.47]
Education level						
Below bachelor’s degree	ref		ref		ref	
Bachelor’s degree	0.91	[0.46, 1.81]	0.91	[0.69, 1.21]	1.20	[0.88, 1.62]
Postgraduate degree	1.87	[0.95, 3.68]	0.63	[0.40, 0.98]*	0.84	[0.48, 1.48]
Clinical position						
Physician	ref		ref		ref	
Nurse	1.47	[0.47, 4.54]	1.45	[0.95, 2.20]	0.81	[0.55, 1.19]
Paramedic	2.19	[0.65, 7.34]	0.79	[0.53, 1.18]	0.64	[0.42, 0.96]
Managerial position						
No	ref		ref		ref	
Yes	0.72	[0.27, 1.92]	1.43	[0.87, 2.33]	0.84	[0.51, 1.37]
Teaching position						
No	ref		ref		ref	
Yes	0.91	[0.22, 3.70]	0.99	[0.74, 1.32]	1.18	[0.79, 1.78]

*AOR *adjusted odds ratio *95%CI *95% confidence intervals

^*^p < 0.05; †*p* < 0.01

As a sensitivity analysis, a mixed-effects logistic regression model was used to capture differences across countries. The results aligned with those of the country-specific logistic regression models, indicating that higher scores in the domain of changes in work and daily routines were associated with elevated A&D levels across the three countries. Conversely, higher scores in the attitudes domain were linked to lower levels of A&D in both Indonesia and Taiwan (Appendix Table S2). Further analysis explored the associations between specific COVID-19-related changes in work and daily routines and the manifestation of A&D symptoms. Notably, the effects of factors such as transportation, work-related dynamics, family time, dietary habits, and income level—but not leisure time—on A&D varied significantly across the three countries (Appendix Table S3).

## Discussion

In this cross-country study, we investigated the potential associations of A&D with COVID-19-related knowledge, attitudes, and practices among HCPs in Taiwan, Indonesia, and Vietnam. Our findings revealed significant variations in A&D levels and their correlations across these three countries, highlighting the complex interplay of pandemic-related factors on mental health among healthcare workers. Numerous studies have evaluated the effects of COVID-19 on the KAP of HCPs. Most studies revealed a commendable understanding of COVID-19 among HCPs. A systematic review assessing the COVID-19-related KAP of HCPs revealed good knowledge (72.2%), a positive attitude (70.9%), and adept practices (78.8%) [44]. These findings are similar to ours. Substantial between-country variations have also been reported in these parameters, possibly stemming from differences in study populations and sample sizes. By using standardized data collection and research methods, we revealed high knowledge levels in HCPs in Taiwan and relatively low knowledge and attitude levels in those in Indonesia. A study demonstrated that > 70% of HCPs had positive attitudes toward general infection control measures [45]; similarly, our study revealed favorable attitudes and behaviors among most HCPs. In a systematic review and meta-analysis, 78.8% of all HCPs adhered to COVID-19-related infection control measures [44]. These insights collectively shape our understanding of global disparities in the COVID-19-related KAP of HCPs.

The COVID-19 pandemic compromised the mental well-being of frontline HCPs worldwide. A comprehensive systematic review and meta-analysis revealed that among HCPs providing medical services during the pandemic, the predominant psychological response was fear, followed by burnout, A&D, and stress [46]. The review further indicated that more than two-thirds of HCPs experienced COVID-19-related fear: the prevalence of fear ranged from 67% [47] to 77.1% [48]. Although previous studies assessed A&D using diverse measurement tools—including the 2-item and 9-item Patient Health Questionnaire (PHQ-2 & PHQ-9), Depression-Anxiety-Stress Scale-21 (DASS-21), and Hospital Anxiety and Depression Scale 14 items (HADS), among others—several common factors were consistently associated with the elevated prevalence of fear and A&D among HCPs. A key factor was the perceived inability to ensure adequate patient care because of the challenges pertaining to the limited availability of medical resources and the shortage of personnel to cope with the sudden surge in COVID-19 cases [49]. Another factor was the possibility of bringing the virus home and infecting family and friends; this fear was coupled with that of societal stigma [50]. The anxiety levels of HCPs might have increased further because of the fear of personal infection and the subsequent need for isolation, which could translate into a shortage of HCPs on the COVID-19 front line [51]. Additionally, the insufficient PPE supply and unfamiliarity with PPE use, particularly in the initial stages of the pandemic, might have contributed to the development of fear and A&D in HCPs. Furthermore, HCPs might not have received timely training in COVID-19 prevention and control protocols, which resulted in increased risks of infection and mortality [52, 53].

In this study, the associations of A&D with COVID-19-related knowledge and attitudes, experiences related to working conditions and PPE availability during the pandemic, and COVID-19-related changes in work and daily routines varied across the three countries. Notably, a reduction in the A&D disorder was associated with an increase in COVID-19-related knowledge and attitudes among HCPs in Taiwan. Improvements in working conditions and PPE availability were unexpectedly associated with an increased risk of A&D among HCPs in Indonesia. COVID-19-related changes in work and daily routines significantly influenced A&D in HCPs in three countries. The observed variations in A&D across countries may be attributable to the complex interplay among the pandemic, control measures, healthcare systems, and cultural nuances; this necessitates in-depth investigations in the future. Our multinational comparison revealed a high prevalence of A&D among HCPs during the pandemic. This prevalence was significantly higher among HCPs in Taiwan than those among those in Indonesia or Vietnam. These findings are consistent with those of studies reporting that more than two-thirds of all HCPs experience anxiety [54]. Despite Taiwan’s exemplary performance in healthcare resource allocation and pandemic control, HCPs may remain susceptible to psychological distress. Therefore, monitoring system implementation in healthcare facilities and developing effective interventions for HCPs are crucial for enhancing the resilience of these professionals. This can be achieved by ensuring healthy work environments, fostering positive attitudes, and maintaining harmonious relationships among HCPs and between HCPs and patients, thereby improving the quality of care for both patients with COVID-19 and those without it [55]. Additional attention and efforts are needed to assess the long-term psychological effects of COVID-19 on the mental health of HCPs, such as physicians, nurses, and medical staff.

### Strengths and limitations

The strength of this study lies in the standardized methodology and questionnaire used for data collection. However, this study has several limitations that must be acknowledged. First, the cross-sectional nature of this study prevented us from establishing any causal relationships. Second, data were collected using an online questionnaire; thus, the possibility of a recruitment bias cannot be ignored because individuals who were unable or unwilling to participate in the online survey could not be included in this study. Moreover, the online survey format may have particularly disadvantaged older healthcare workers who may have limited digital literacy or access to technology, potentially excluding an important demographic from the study. Also, we acknowledge that the use of different survey platforms may have introduced potential response bias due to factors such as participants' trust in data security or familiarity with the platform interface. To address this, we implemented standardized questionnaire content, reassurances of confidentiality and anonymity, and relied on trusted dissemination channels. While these efforts aimed to mitigate bias, the possibility cannot be entirely excluded. Future research could further explore how platform choice influences response behavior, particularly in multinational contexts. Third, while our study provides valuable insights into anxiety and depression among healthcare professionals across Taiwan, Indonesia, and Vietnam, it is essential to acknowledge the temporal variations in data collection. The surveys were conducted during different periods: December 2021 to February 2022 in Taiwan, February 2021 to June 2021 in Indonesia, and July 2021 to February 2022 in Vietnam. These timing differences reflect the administrative challenges inherent in cross-national research. Each country experienced distinct COVID-19 epidemiological scenarios during these periods: Taiwan was managing its initial Omicron wave with a highly successful previous containment strategy, Indonesia was navigating the Delta and early Omicron variant outbreaks with significant community transmission, and Vietnam was transitioning from strict zero-COVID policies to more adaptive management strategies. These contextual variations could potentially influence healthcare professionals' mental health experiences, as the pandemic's progression, local virus variants, healthcare system pressures, and national response strategies differed significantly. Despite these temporal disparities, our multi-country approach provides a comparative perspective on mental health challenges faced by healthcare workers during different stages of the pandemic. We recognize that the timing differences might introduce some heterogeneity in our results, and thus, our findings should be interpreted with careful consideration of the specific epidemiological and healthcare contexts of each country during their respective survey periods. Fourth, the use of self-report questionnaires may introduce a reporting bias or an inaccuracy bias. Also, since we were unable to collect information on participants' mental health history, individuals with pre-existing anxiety or depression may have been included, which could influence the observed associations. Finally, we used a nonrandom sampling method, in which Taiwanese HCPs were recruited from a single hospital, Vietnamese HCPs were recruited from two hospitals in northern Vietnam, and Indonesian HCPs were recruited from three hospitals in Jakarta. While all participating institutions were large medical centers, this sampling strategy inherently constrains the generalizability of our findings. Readers should interpret our results with caution, recognizing the potential sampling limitations that may impact the broader representativeness of our research. Further validation through large-scale longitudinal studies is imperative to enhance the robustness and generalizability of our results.

## Conclusion

By examining between-country variations in HCPs’ COVID-19-related knowledge, attitudes, and practices, we explored their responses to the COVID-19 pandemic. Our findings revealed noteworthy correlations between COVID-19-related factors and A&D in HCPs, providing insights into the diverse responses among HCPs in different countries. Furthermore, we revealed the effects of experiences related to working conditions and PPE availability during the pandemic and COVID-19-related changes in work and daily routines on the A&D symptoms in HCPs, highlighting the need for monitoring the short- and long-term effects of COVID-19-related factors on the mental health of HCPs. Furthermore, the observed country-specific associations indicate the need for customized support mechanisms and tailored interventions for HCPs within specific policy and country contexts. Nuanced and context-specific investigations are warranted to address the distinct psychological needs of HCPs worldwide in the post-COVID-19 era.

## Supplementary Information

Supplementary Material 1.

## Data Availability

The datasets generated and/or analyzed during the current study are not publicly available due the regulation of the Institutional Review Board of Taipei Medical University but are available from Ya-Wen Chiu on reasonable request.
