# Supplementary material for: Anxiety, depression, and their associations with COVID-19-related knowledge, attitudes, and practices among healthcare professionals: a multinational cross-sectional survey
Source: BMC Psychol. 2025 May 2;13:463. doi: 10.1186/s40359-025-02783-0 (PMC12049025; doi:10.1186/s40359-025-02783-0)
Supplement: Supplementary file 1 — Supplementary Material 1. [file 40359_2025_2783_MOESM1_ESM.docx]

**Supplementary**

**Fig. S1. Box plot of six COVID-19-related domains by countries**


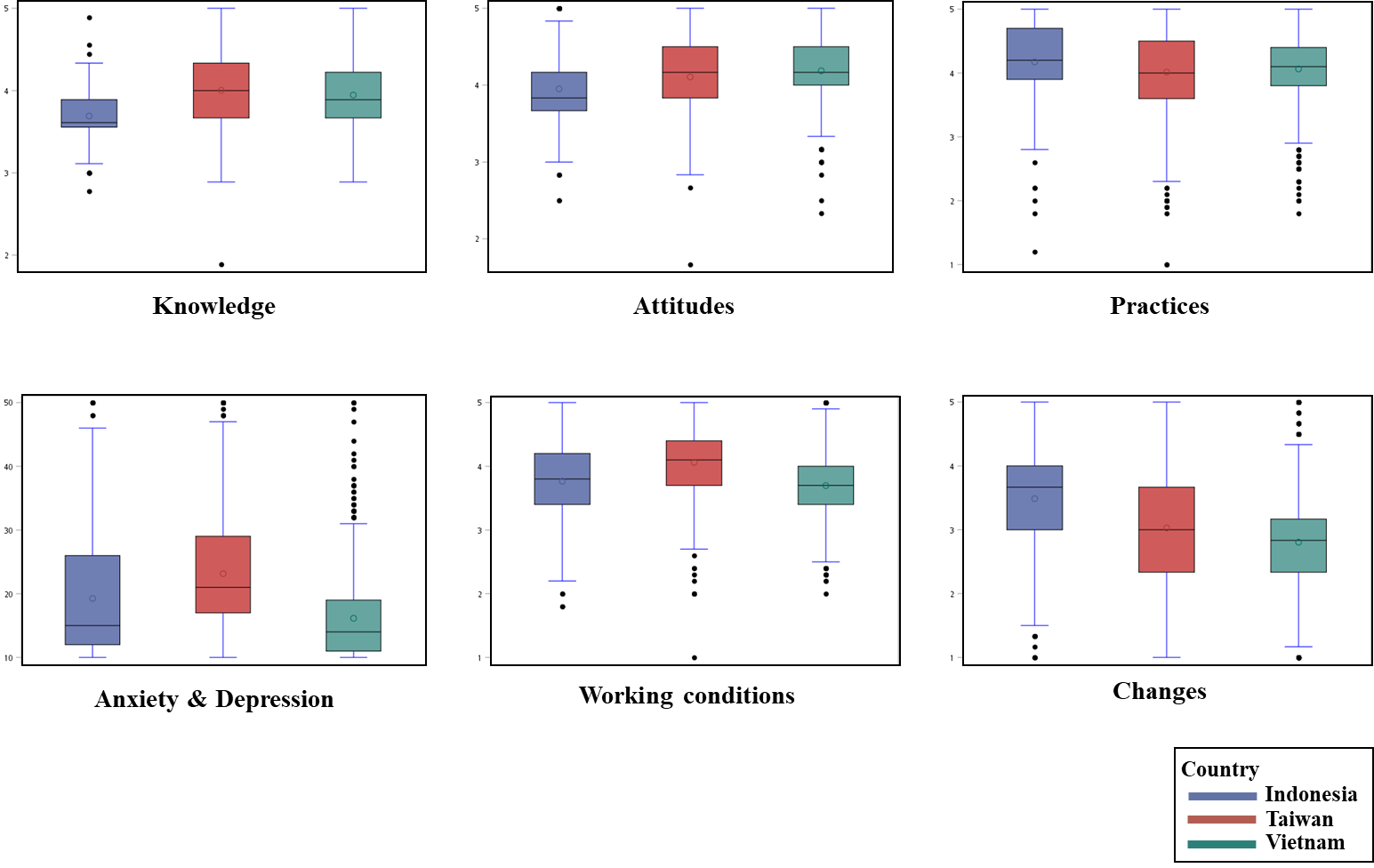


**Table S1. Correlation matrix of anxiety and depression and five COVID-19-related domains**

| Specific domains | Anxiety and depression | Knowledge | Attitudes | Practices | Working conditions | Changes in work and daily routines |
| --- | --- | --- | --- | --- | --- | --- |
| Anxiety and depression | 1.0 | -0.04 | -0.12 | 0.01 | 0.17 | 0.36 |
| Knowledge |  | 1.0 | 0.55 | 0.19 | 0.20 | 0.01 |
| Attitudes |  |  | 1.0 | 0.30 | 0.20 | 0.02 |
| Practices |  |  |  | 1.0 | 0.38 | 0.15 |
| Working conditions |  |  |  |  | 1.0 | 0.15 |
| Changes in work and daily routines |  |  |  |  |  | 1.0 |

**Table S2. Mixed-effects logistic regression model** **for the country specific effects of five COVID-19-related domains on anxiety and depression**

| **Domains** | **Indonesia (n=402)** | | **Taiwan (n=1,645)** | | **Vietnam (n=1,476)** | |
| --- | --- | --- | --- | --- | --- | --- |
|  | AOR | 95%CI | AOR | 95%CI | AOR | 95%CI |
| **Knowledge (K)** |  |  |  |  |  |  |
| Q1 (K < 3.66) | ref |  | ref |  | ref |  |
| Q2 (3.66 ≦ K < 3.88) | 0.97 | [0.55, 1.71] | 0.77 | [0.44, 1.34] | 1.11 | [0.64, 1.93] |
| Q3 (3.88 ≦ K < 4.22) | 1.11 | [0.51, 1.83] | 0.63 | [0.30, 1.35] | 1.26 | [0.59, 2.69] |
| Q4 (K ≧ 4.22) | 0.75 | [0.21, 1.07] | 0.46 | [0.15, 1.44] | 1.31 | [0.42, 4.09] |
| **Attitudes (A)** |  |  |  |  |  |  |
| Q1 (A < 3.83) | ref |  | ref |  | ref |  |
| Q2 (3.83 ≦ A < 4.16) | 0.78 | [0.62, 0.70]* | 0.78 | [0.62, 0.99]* | 0.78 | [0.62, 0.99]* |
| Q3 (4.16 ≦ A < 4.5) | 0.61 | [0.36, 0.50]* | 0.47 | [0.28, 0.79]* | 0.66 | [0.39, 1.11] |
| Q4 (A ≧ 4.5) | 0.66 | [0.34, 0.63]* | 0.43 | [0.23, 0.80]* | 0.66 | [0.36, 1.23] |
| **Practices (P)** |  |  |  |  |  |  |
| Q1 (P < 3.7) | ref |  | ref |  | ref |  |
| Q2 (3.7 ≦ P < 4.1) | 0.94 | [0.70, 1.04] | 0.93 | [0.69, 1.24] | 0.89 | [0.66, 1.19] |
| Q3 (4.1 ≦ P < 4.5) | 0.84 | [0.67, 0.81]* | 0.84 | [0.67, 1.06] | 0.84 | [0.67, 1.06] |
| Q4 (P ≧ 4.5) | 0.78 | [0.49, 0.77]* | 1.02 | [0.65, 1.58] | 0.95 | [0.61, 1.48] |
| **Working conditions (W)** |  |  |  |  |  |  |
| Q1 (W < 3.5) | ref |  | ref |  | ref |  |
| Q2 (3.5 ≦ W < 3.9) | 1.24 | [0.98, 1.71] | 1.24 | [0.98, 1.56] | 1.24 | [0.98, 1.56] |
| Q3 (3.9 ≦ W < 4.3) | 1.00 | [0.79, 1.13] | 1.00 | [0.79, 1.27] | 1.00 | [0.79, 1.27] |
| Q4 (W ≧ 4.3) | 1.13 | [0.80, 1.53] | 1.01 | [0.71, 1.43] | 1.09 | [0.77, 1.54] |
| **Changes in work and daily routines (C)** |  |  |  |  |  |  |
| Q1 (C < 2.3) | ref |  | ref |  | ref |  |
| Q2 (2.3 ≦ C < 3.0) | 2.13 | [1.36, 5.51]* | 1.87 | [1.22, 2.86]* | 2.36 | [1.54, 3.62]* |
| Q3 (3.0 ≦ C < 3.5) | 3.13 | [2.45, 10.60]* | 3.13 | [2.45, 4.00]* | 3.13 | [2.45, 4.00]* |
| Q 4 (C ≧ 3.5) | 4.08 | [3.18, 17.87]* | 4.08 | [3.18, 5.23]* | 4.08 | [3.18, 5.23]* |
| Note: *p<0.05; AOR: Adjusted Odds Ratio (adjusted for covariates, including sociodemographic characteristics and work positions) | | | | | | |

**Table S3. Multivariate analysis for the associations of A&D level with COVID-19-related changes in work and daily routines**

| **Variables** | **Indonesia (n=402)** | | **Taiwan (n=1,645)** | | **Vietnam (n=1,476)** | |
| --- | --- | --- | --- | --- | --- | --- |
|  | **AOR** | **95% CI** | **AOR** | **95% CI** | **AOR** | **95% CI** |
| **Transportation, routine travel** |  |  |  |  |  |  |
| < 3 points | ref |  | ref |  | ref |  |
| ≧ 3 points | 1.80 | [0.97, 3.35] | 1.35 | [1.03, 1.76]* | 1.69 | [1.25, 2.27] † |
| **Work** |  |  |  |  |  |  |
| < 3 points | ref |  | ref |  | ref |  |
| ≧ 3 points | 1.25 | [0.50, 3.11] | 1.39 | [1.05, 1.84]* | 1.93 | [1.29, 2.88] † |
| **Leisure time** |  |  |  |  |  |  |
| < 3 points | ref |  | ref |  | ref |  |
| ≧ 3 points | 1.77 | [0.89, 3.49] | 0.73 | [0.55, 1.03] | 1.13 | [0.86, 1.50] |
| **Family time** |  |  |  |  |  |  |
| < 3 points | ref |  | ref |  | ref |  |
| ≧ 3 points | 0.55 | [0.27, 1.14] | 1.61 | [1.18, 2.19] † | 0.62 | [0.44, 0.89]* |
| **Dietary habit** |  |  |  |  |  |  |
| < 3 points | ref |  | ref |  | ref |  |
| ≧ 3 points | 1.16 | [0.39, 3.48] | 1.25 | [0.92, 1.70] | 1.61 | [1.12, 2.33]* |
| **Income** |  |  |  |  |  |  |
| < 3 points | ref |  | ref |  | ref |  |
| ≧ 3 points | 3.20 | [1.14, 8.99]* | 1.64 | [1.25, 2.16] † | 1.32 | [0.93, 1.87] |
| Note: *p<0.05; †p<0.01; AOR: Adjusted Odds Ratio (adjusted for covariates, including sociodemographic characteristics and work positions) | | | | | | |
